# Extending KIDs to the Mid-IR for Future Space and Suborbital Observatories

**DOI:** 10.1007/s10909-020-02364-y

**Published:** 2020-02-14

**Authors:** J. Perido, J. Glenn, P. Day, A. Fyhrie, H. Leduc, J. Zmuidzinas, C. McKenney

**Affiliations:** 1 Department of Astrophysics and Planetary Sciences, University of Colorado at Boulder, Boulder, CO 80309 USA; 2 NASA Jet Propulsion Laboratory (JPL), Pasadena, USA; 3 California Institute of Technology, Pasadena, USA

**Keywords:** Kinetic inductance detector, 10 micron, Mid-infrared, Far-infrared, Astrophysics

## Abstract

The galaxy evolution probe (GEP) is a concept for a probe-class space observatory to study the physical processes related to star formation over cosmic time. To do so, the mid- and far-infrared (IR) spectra of galaxies must be studied. These mid- and far-IR observations require large multi-frequency arrays, sensitive detectors. Our goal is to develop low NEP aluminum kinetic inductance detectors (KIDs) for wavelengths of 10–400 ${\upmu }{{\hbox {m}}}$ for the GEP and a pathfinder long-duration balloon (GEP-B) that will perform precursor GEP science. KIDs for the lower wavelength range (10–100 ${\upmu }{{\hbox {m}}}$) have not been previously implemented. We present an absorber design for KIDs sensitive to wavelengths of 10 ${\upmu }{{\hbox {m}}}$ shown to have around 75–80% absorption efficiency through ANSYS HFSS (high-frequency structure simulator) simulations, challenges that come with optimizing our design to increase the wavelength range, initial tests on our design of fabricated 10 ${\upmu }{{\hbox {m}}}$ KIDs, and theoretical NEP calculations.

## Introduction

Observations of the mid- and far-infrared spectra of galaxies are a necessary means to explore the origins of galaxies, stars, and planets that make up the universe. From these spectra, the correlations of star-formation rates with other physical properties can be investigated to expand our understanding of galaxy evolution. Galaxies have historically been studied through the emission features of dust and atomic fine structure lines in the far-IR. However, another known and studied region rich with information lies in the rest-frame emission features of polycyclic aromatic hydrocarbons (PAHs) with emission bands in the 3–13 ${\upmu }{{\hbox {m}}}$ range [1]. PAHs can be used to measure redshifts and characterize interstellar physical conditions and chemistry in millions of galaxies with a new infrared space observatory. The galaxy evolution probe (GEP) is a concept for a probe-class space observatory, with the goal to detect galaxies in these mid- and far-IR regions by utilizing large arrays of back-illuminated, lumped-element, microlens-coupled, sensitive aluminum kinetic inductance detectors (KIDs).

Although KIDs research is a popular and growing field, KIDs for detection at wavelengths from 10–100 ${\upmu }{{\hbox {m}}}$ have not been an area of particular emphasis. The investigation for KIDs at these wavelengths is also useful for other space observatories, such as the Origins Space Telescope. To address this, we investigate KID absorber designs capable of absorbing at GEP’s shortest wavelength ($=10 {\upmu }{{\hbox {m}}}$) while achieving background limited sensitivity.

The GEP will have one instrument with two modules, an imager (GEP-I), and a dispersive spectrometer (GEP-S) consisting of 25,735 detectors and 24,640 detectors, respectively. The sensitivity requirements for the modules are a NEP of $1 \times 10^{{-18}}$ W/${\sqrt{\text {Hz}}}$ for the GEP-I and $1 \times 10^{-19}$ W/${\sqrt{\text {Hz}}}$ for the GEP-S. We also plan to demonstrate performance and raise the detector technology readiness level (TRL) with a pathfinder long-duration balloon (GEP-B) that will perform precursor GEP science. The KIDs on GEP-B will need a NEP on the order of $10^{-16}$ W/${\sqrt{\text {Hz}}}$. The increase in the NEP when compared to the GEP is due to the (ambient) temperatures of the optical components on the GEP-B.

In this paper, we will present ANSYS HFSS^1^ (high-frequency structure simulator) software suite simulations of KID absorber designs capable of absorbing at 10 ${\upmu }{{\hbox {m}}}$ and greater, preliminary measurements of the critical temperature of a fabricated test device and evidence of two-level system (TLS) noise, and theoretical NEP calculations for GEP.

## MID-IR KID Designs and HFSS Simulations

**Fig. 1:**
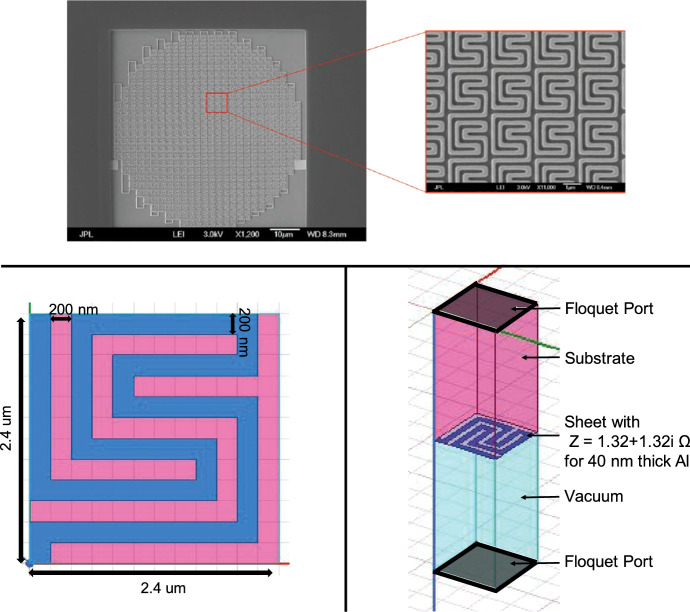
*Top* Photograph of the inductor portion of a 10 ${\upmu }{{\hbox {m}}}$ KID. The unit cell shown in Fig. 1*Bottom Left* is repeated to cover the entire absorber area, which has a circular envelope with a diameter of 60 ${\upmu }{{\hbox {m}}}$. *Bottom Left* short-meander unit cell geometry. The blue represents Al with $Z = (1.32 + 1.32i){\Omega }$ and the pink represents the Si substrate. The material of the microlens array that couples radiation to the KIDs will be made of the same material as the substrate. The total unit cell size depicted is $2.4 \times 2.4{\upmu }{{\hbox {m}}}$, but will vary according to wavelength and substrate material. *BottomRight* 3D model of HFSS unit cell simulation. The plane wave travels from the top Floquet port to the bottom Floquet Port. The vacuum and substrate box heights are not to scale. The heights were adjusted to show where the Floquet ports are assigned. The actual box height is equal to two times the longest wavelength of the simulation frequency sweep (Color figure online)

The inductors will have the unit cell repeated in a circular envelope. This is shown in Fig. 1*Top* where the geometry in Fig. 1*Bottom Left* is repeated to form the absorber. To evaluate the absorption of the inductors at 10 ${\upmu }{{\hbox {m}}}$ ($\nu = 30$ THz), we used ANSYS HFSS. Simulations were done using a single unit cell simulated in an infinite periodic grid to approximate the repeated geometry across the absorber. A single unit cell is shown in Fig. 1*Bottom Right*. Floquet ports were assigned to the faces of the vacuum and substrate boxes which allows simulation of a plane wave with two Floquet modes that represent the incident horizontally and vertically polarized electromagnetic plane waves. HFSS achieves the periodic grid condition by assigning linked boundaries between parallel walls of the vacuum and substrate boxes of the unit cell. The linked boundaries enforce the parallel walls to have the same fields. The aluminum (blue meander in Fig. 1*Bottom*) was modeled with sheets of impedance set to that of 40-nm-thick aluminum exposed to 30 THz electromagnetic radiation on a substrate (Fig. 1*Bottom*). The impedance was determined by the following equation:

1 $$\begin{aligned} Z_s = \frac{1+i}{\delta \sigma }\coth \Big (\frac{1+i}{\delta }t\Big ), \end{aligned}$$

where $\delta = 11.14 \ {{\text {nm}}}$ is the skin depth, $\sigma = 6.87 \times 10^{7} \ \Omega ^{-1}{{\mathrm {m}}}^{-1}$ is the conductivity, and *t* is the thickness of the aluminum [2]. Between the different geometries investigated, two showed promising results: one was a short meander (Fig. 1*Bottom Left*) and the other a dual polarization geometry (Fig. 3*Left*).

**Fig. 2:**
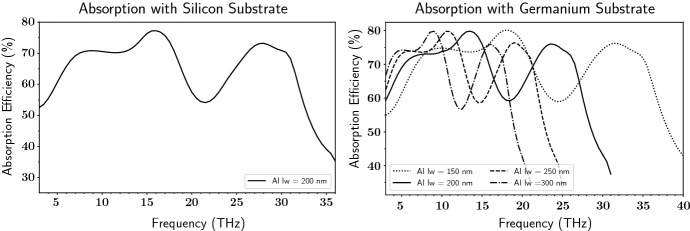
HFSS-simulated absorption efficiency of short meander (lw = aluminum line width). *Left* Absorption efficiency of about 73% near 30 THz on Si substrate. *Right* Absorption efficiency shifts to lower frequencies (greater wavelength) as lw is increased on Ge substrate. Absorption efficiency varies from 16–32 THz, showing that the detector is capable of absorbing different wavelengths by adjusting the aluminum line width and unit cell size

**Fig. 3:**
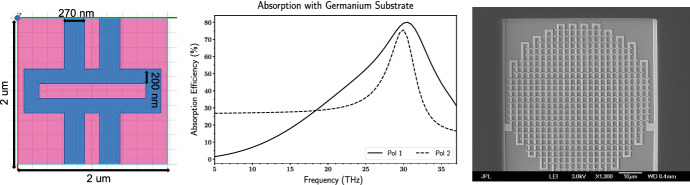
*Left* Dual polarization 10 ${\upmu }{{\hbox {m}}}$ KID inductor geometry. HFSS simulations of this geometry were performed the same as the short meander (Fig. 1*Bottom right*) to approximate the repeated geometry across the circular profile of the inductor. The blue represents Al with $Z = (1.32 + 1.32i){\Omega }$ and the pink represents the Si substrate. *Middle* HFSS-simulated absorption efficiency of   75–80% near 30 THz in both polarizations. These absorption profiles match GEP bandwidths better than those in Fig. 2 [4]. *Right* Photograph of the inductor portion of a 10 ${\upmu }{{\hbox {m}}}$ KID. The unit cell shown in the left panel is repeated to cover the entire absorber area, which has a circular envelope with a diameter of 60 ${\upmu }{{\hbox {m}}}$. Current flows along the vertical direction of the meander (Color figure online)

The $2.4\times 2.4\,{\upmu }{{\hbox {m}}}$ unit cell with the short-meander geometry shown in Fig. 1*Bottom Left* has aluminum line widths and gaps of 200 nm. The vertical lines couples to vertically polarized light, and the horizontal portion of the meander increases the resistance per unit length, enabling high absorption efficiency. This geometry was chosen because of its high HFSS-simulated absorption efficiency at 30 THz (Fig. 2*Left*). With aluminum line widths of 200 nm and on a silicon (Si) substrate, the absorption efficiency at 30 THz is near 73%. Since the absorption profile is broad, spectral bands will be defined with band-pass filters. The short meander was also simulated on a germanium (Ge) substrate (Fig. 2*Right*), because Ge has better transmittance than Si from 10–20 ${\upmu }{{\hbox {m}}}$ [3]. The Ge simulations show that increasing the line width which in turn increases the unit cell size shifts the absorption to longer wavelengths. This means different geometries are not needed for different wavelength absorption. However, for absorption at 30 THz on Ge, the aluminum line widths need to be less than 200 nm (Fig. 2*Right*). Without e-beam lithography fabrication of a device with such thin line widths is difficult, so a different geometry was explored, the dual polarization geometry shown in Fig. 3*Left*.

The dual polarization geometry absorbs in both vertical and horizontal polarizations (Fig. 3*Middle*). Another nice feature of this design is a higher absorption efficiency of 75–80% at 30 THz. It absorbs at 30 THz with vertical line widths of 270 nm, horizontal line widths of 200 nm, and 200 nm gaps in a $2\times 2\,{\upmu }{{\hbox {m}}}$ unit cell. Since the unit cell size does not need to scale with aluminum line width there is more room to explore how changing parameters such as line and gap thickness changes the absorption peak profile. This absorber would be useful for observatories that need detectors which absorb in both polarizations.

## Preliminary Measurements

A test device with the short-meander design and 40-nm-thick aluminum was fabricated at the Microdevices Laboratory at JPL, on a Si substrate (Fig. 1*Top*). Each KID consists of a lithographically patterned absorbing inductive section connected to a large interdigitated capacitor and coupled to the feedline with small interdigitated capacitors [5]. The inductor portion of the lumped-element KIDs consists of the short-meander pattern shown in Fig. 1*Bottom Left* repeated across a circular profile with a diameter of 60 ${\upmu }{{\hbox {m}}}$ for effective optical coupling to a microlens array. Initial testing showed over 94% yield for resonant frequencies from 1.4–2 GHz with high internal Q-factors of over 200,000. We performed preliminary measurements to estimate the $T_{{\mathrm{c}}}$ of the device. Evidence of TLS noise was found.

### $T_{{\mathrm{c}}}$ Measurements

**Fig. 4:**
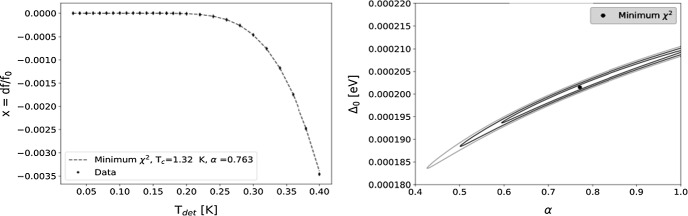
*Left* Fractional frequency shift as a function of temperature. The dashed line represents the best fit model. *Right*$\varDelta \chi ^2$ contour plots of 1$\sigma$, 2$\sigma$, and 3$\sigma$ uncertainties in the parameters. The 1$\sigma$ bounds were used to assign an error to $T_{{\mathrm{c}}}$, which yielded $T_{{\mathrm{c}}} = (1.32 \pm 0.05)$ K

The critical temperature of the test device was estimated by obtaining the fractional frequency shift of a detector’s resonance with increasing temperature from 25 to 400 mK (Fig. 4*Left*). The data were fit to the following equation for fractional frequency shift:

2 $$\begin{aligned} X = \frac{f_{{\mathrm{r}}}(T)-f_{{\mathrm{r}}}(T_{{{\mathrm{base}}}})}{f_{{\mathrm{r}}}(T_{{{\mathrm{base}}}})} = 0.5\alpha \Big (\frac{\sigma _2-\max (\sigma _2)}{\max (\sigma _2)}\Big ), \end{aligned}$$

where $\sigma _2 = \frac{\pi \varDelta _0}{hf_{{\mathrm{r}}}}\Big (1 - \sqrt{\frac{2\pi k_{{\mathrm{b}}}T}{\varDelta _0}}e^{\frac{-\varDelta _0}{k_{{\mathrm{b}}}T}}-2e^{\frac{-\varDelta _0}{k_{{\mathrm{b}}}T}}J_0\Big (\frac{hf}{2k_{{\mathrm{b}}}T}\Big )\Big )$, $f_{{\mathrm{r}}}$ is the resonant frequency, *T* is the temperature, $T_{{{\mathrm{base}}}}$ is the initial temperature, and $k_{\rm B}$ is Boltzmann’s constant [6, 7]. The best fit was determined using the $\chi ^2$ minimization method with $\alpha$ (kinetic inductance fraction) and $\varDelta _0$ (band gap energy) as the estimate parameters. $T_{{\mathrm{c}}}$ was then estimated using the relation $\varDelta _0$=1.764$k_{\rm B}T_{{\mathrm{c}}}$, yielding $T_{{\mathrm{c}}}$ = (1.32 ± 0.05) K. This result is compatible with the $T_{{\mathrm{c}}}$ of aluminum found in the literature [8, 9].

### TLS Noise

**Fig. 5:**
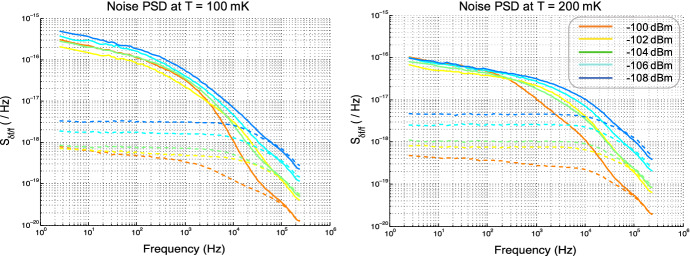
Plots showing evidence of TLS noise. *Dashed lines* = amplitude noise, *solid lines* = phase noise. As the driving power decreases, the noise increases (driving power starts at $-100$ dBm and is attenuated by 2 dB with each curve). At higher temperature (200 mK), the noise decreases (Color figure online)

TLS noise is caused by an ensemble of switching dipoles in the substrate that fluctuates the dielectric constant, thereby altering the capacitance of the KIDs [10]. Evidence of TLS noise can be detected from the noise power spectral densities (PSDs) [7]. As the temperature is increased, TLS noise should decrease, and as the driving power increases, TLS noise should also decrease, because the two-level systems become saturated in the upper state [7]. Figure 5 shows noise PSDs at $T = 100$ mK and $T = 200$ mK. We see there is an evident decrease in phase noise at the higher temperature. With drive power attenuation, the noise increases which is another characteristic of TLS noise shown in Fig. 5.

## GEP Theoretical NEP

**Fig. 6:**
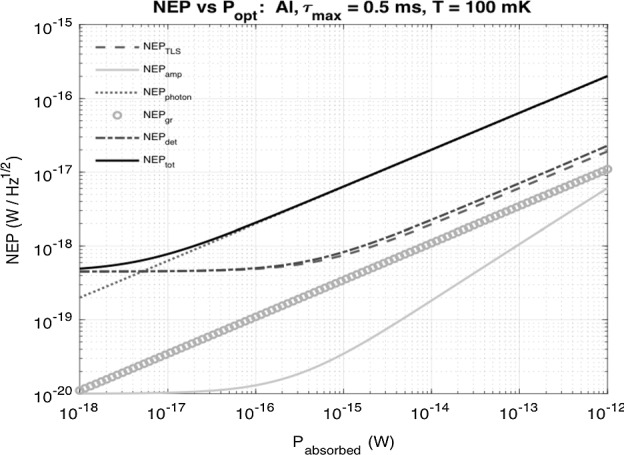
Theoretical NEP plot for GEP

Since we have not yet done optical testing, we provide a theoretical NEP calculation for GEP that takes into account the measured TLS noise and estimated $T_{{\mathrm{c}}}$ and $\alpha$ from Sect. 3. The total NEP of the test device is calculated by adding all NEP contributions in quadrature, $\ {{\mathrm{NEP}}}_{{{\mathrm{tot}}}}^2 = {{\mathrm{NEP}}}_{{{\mathrm{TLS}}}}^2 + {{\mathrm{NEP}}}_{{{\mathrm{amp}}}}^2 + {{\mathrm{NEP}}}_{{{\mathrm{photon}}}}^2 + {{\mathrm{NEP}}}_{{{\mathrm{GR}}}}^2$ [6, 7]. Since we have not measured quasiparticle lifetimes ($\tau _{{{\mathrm{qp}}}}$), an assumption was made based on previously measured 40-nm Al KIDs that $\tau _{{{\mathrm{qp}}}} = 500 \ {\upmu s}$ [11]. The operating temperature will be $T=100\ {{\text {mK}}}$ for GEP. At 10 ${\upmu }{{\hbox {m}}}$ , we expect the photon loading to be $2.5 \times 10^{-15}$ W. This corresponds to a theoretical NEP of $1 \times 10^{-17}$ W/${\sqrt{\text {Hz}}}$ (Fig. 6).

## Summary and Future Work

We have designed aluminum KIDs on Si and Ge substrates with meanders for tuning resistivity that yield simulated absorption efficiencies of 73–80% at 10 ${\upmu }{{\hbox {m}}}$. The absorption can be shifted in wavelength by adjusting the aluminum line widths and unit cell sizes of the geometry repeated across the inductor of the KID. A test device with 10 ${\upmu }{{\hbox {m}}}$ aluminum KIDs was fabricated, from which $T_{{\mathrm{c}}} = (1.32 \pm 0.05)$ K was estimated and evidence of TLS noise was found. We also present a theoretical NEP for GEP at 10 ${\upmu }{{\hbox {m}}}$ that takes into account the estimated $T_{{\mathrm{c}}}$ and measured TLS noise from the fabricated device.

Our next steps are to investigate zinc sulfide as an anti-reflection coating in the HFSS simulations to increase absorption efficiency [3], couple the KIDs with the short-meander geometry to a microlens array for optical testing, fabricate a device with the dual polarization geometry, and measure $\tau _{{{\mathrm{qp}}}}$ and NEPs for both. We will also begin development of 30 ${\upmu }{{\hbox {m}}}$ aluminum KIDs.

## Footnotes

1 https://www.ansys.com/products/electronics/ansys-hfss
